# Active Life for Brain Health: A Narrative Review of the Mechanism Underlying the Protective Effects of Physical Activity on the Brain

**DOI:** 10.3389/fnagi.2021.761674

**Published:** 2021-11-30

**Authors:** Hiroyuki Umegaki, Takashi Sakurai, Hidenori Arai

**Affiliations:** ^1^Department of Community Healthcare and Geriatrics, Nagoya University Graduate School of Medicine, Nagoya, Japan; ^2^Center for Comprehensive Care and Research on Memory Disorders, National Center for Geriatrics and Gerontology, Obu, Japan; ^3^National Center for Geriatrics and Gerontology, Obu, Japan

**Keywords:** Alzheimer’s disease, brain-derived neurotrophic factor, exercise, dementia, neurodegeneration, physical activity, white matter

## Abstract

A growing body of evidence clearly indicates the beneficial effects of physical activity (PA) on cognition. The importance of PA is now being reevaluated due to the increase in sedentary behavior in older adults during the COVID-19 pandemic. Although many studies in humans have revealed that PA helps to preserve brain health, the underlying mechanisms have not yet been fully elucidated. In this review, which mainly focuses on studies in humans, we comprehensively summarize the mechanisms underlying the beneficial effects of PA or exercise on brain health, particularly cognition. The most intensively studied mechanisms of the beneficial effects of PA involve an increase in brain-derived neurotrophic factor (BDNF) and preservation of brain volume, especially that of the hippocampus. Nonetheless, the mutual associations between these two factors remain unclear. For example, although BDNF presumably affects brain volume by inhibiting neuronal death and/or increasing neurogenesis, human data on this issue are scarce. It also remains to be determined whether PA modulates amyloid and tau metabolism. However, recent advances in blood-based biomarkers are expected to help elucidate the beneficial effects of PA on the brain. Clinical data suggest that PA functionally modulates cognition independently of neurodegeneration, and the mechanisms involved include modulation of functional connectivity, neuronal compensation, neuronal resource allocation, and neuronal efficiency. However, these mechanisms are as yet not fully understood. A clear understanding of the mechanisms involved could help motivate inactive persons to change their behavior. More accumulation of evidence in this field is awaited.

## Introduction

Cognitive decline and dementia are major health concerns worldwide. A major cause of dementia is Alzheimer’s disease (AD) and very recently pharmacological treatment with aducanumab was approved in the United States for AD. However, the effects of this drug are far from a “cure” and other therapeutics are needed.

Cumulative research results clearly indicate the beneficial effects of physical activity (PA) on brain health, and some reports have suggested that physical inactivity in older adults caused by COVID-19 pandemic-related lockdowns have had a negative impact on brain health (Fiorenzato et al., 2021; Ismail et al., 2021; Tondo et al., 2021; Znazen et al., 2021).

Physical activity has been found to have a positive impact on cognition in a wide range of individuals, from those who are cognitively normal to those who have dementia (Heyn et al., 2004; Aarsland et al., 2010; Sofi et al., 2011; Groot et al., 2016). Many basic, epidemiological, and observational cohort studies, as well as randomized controlled studies, have provided evidence of the beneficial effects of PA on cognition (Sofi et al., 2011). Various studies, mainly epidemiological, have also reported that PA reduces the risk of onset of dementia (Hamer and Chida, 2009). However, the mechanism underlying these beneficial effects of PA on brain health has not been completely elucidated. A neurodegenerative process may be accelerated with aging through pathological changes, oxidative stress, or inflammation, and PA may counteract these neurodegenerative processes. PA may prevent the development of brain pathologies, including AD-related and cerebrovascular pathologies, although some evidence indicates that the cognitive benefits of PA are independent of pathological changes in the brain (Buchman et al., 2019). PA may protect the gray and white matter structure of the brain and may also enhance physiological aspects of the brain, including cerebral blood flow and neurotrophic factor release. Functional enhancement of each neuron and the neural network as a whole may also be involved.

This review, which largely focuses on human studies, describes the various mechanisms of the brain-protective effects induced by PA. We divide the beneficial mechanisms of PA or exercise into several components and review the structural, physiological, anti-neurodegenerative, and functional effects of PA or exercise (Table 1).

**TABLE 1:** Hypothetical mechanisms underlying the beneficial effects of PA on brain health.

1. Structural mechanism 1) Brain volume 2) White matter 3) Small vessel disease 2. Physiological mechanism 1) BDNF 2) Cerebral blood flow 3. Anti-neurodegenerative mechanism 1) Amyloid and tau 2) Oxidative stress 3) Inflammation 4. Functional mechanism 1) Functional connectivity 2) Neuronal compensation 3) Neural resource allocation 4) Neuronal efficacy

*W3510PA, physical activity; BDNF, brain-derived neurotrophic factor.*

## Effects on Brain Structure

### Brain Volume

The volume of the brain declines with aging, starting in midlife (Scahill et al., 2003). This reduction in brain volume is presumed to reflect decreases in neuron volume and number (Gogniat et al., 2021). One of the mechanisms underlying the brain-protective effects of PA is the preservation of brain volume, or even an increase in brain volume. Observational studies have found that brain volume, particularly that of the hippocampus, is preserved in more active people (Erickson et al., 2010; Flöel et al., 2010; Maasakkers et al., 2021). PA is also associated with preservation of cortical thickness (Walhovd et al., 2014; Lee et al., 2016).

Many interventional trials have also shown the positive impact of PA on brain volume. Some studies showed that PA interventions reduced the development of brain atrophy compared with controls (Best et al., 2015), and some studies even demonstrated an increase in brain volume after an exercise intervention (Colcombe et al., 2006; Tao et al., 2017) in older adults. A systematic review of the effects of exercise intervention on the hippocampus concluded that aerobic exercise helped to preserve hippocampal volume by preventing its decrease, while some of the included studies even showed an increased volume (Firth et al., 2018). The characteristics of these representative studies are summarized in Table 2.

**TABLE 2:** Brain volume changes associated with PA levels.

	**Participants**	**Study design**	**Finding**
**Observational**			
Maasakkers et al. (2021)	718 community-dwelling older adults (mean age 66)	Cross-sectional	High sedentary levels associated with lower hippocampal volumes
Flöel et al. (2010)	75 community-dwelling older adults (mean age 60.5)	Cross-sectional	Higher PA levels associated with increased cerebral gray matter volume in prefrontal and cingulate cortex
Erickson et al. (2010)	299 participants recruited from a Medicare database (mean age 78)	Longitudinal (9 years)	Higher PA levels associated with greater volumes of frontal, occipital, entorhinal, and hippocampal regions 9 years later
Lee et al. (2016)	1842 participants who attended a preventive medical check-up (mean age 64)	Cross-sectional	PA associated with greater global and frontal mean thickness
Walhovd et al. (2014)	203 community-dwelling older adults (mean age 54)	Longitudinal (3.6 years)	Higher PA levels associated with less thinning of left prefrontal cortex
**Interventional**			
Best et al. (2015)	155 (52 experimental) community-dwelling older women (mean age 70)	Resistance training twice a week for 2 years	Resistance training reduced cortical white matter atrophy
Colcombe et al. (2006)	59 (half experimental) older subjects without neurological defects (mean age 66.5)	Aerobic exercise intervention for 6 months	Significant increases in brain volume, in both gray and white matter regions
Tao et al. (2017)	62 (21 tai chi chuan, 16 Baduanjin) health volunteers (mean age 62)	Tai chi chuan and Baduanjin exercise (60 min for 5 days a week) for 12 weeks	Significant increases in gray matter volume in insula, medial temporal lobe, and putamen after 12 weeks of exercise

*PA, physical activity.*

Although the precise mechanisms remain to be elucidated, the neuroprotective effects of PA may be exerted through increased brain-derived neurotrophic factor (BDNF) and blood flow or reduced oxidative stress and amyloid accumulation (as discussed below). Recently, the MAPT study reported that more physically active individuals had lower blood concentrations of neurofilament light chain, a well-established biomarker of neurodegeneration (Raffin et al., 2021). These results suggest that PA may ameliorate neurodegeneration. Moreover, decades of research have shown that adult neurogenesis persists throughout life, although it declines with aging. Hippocampal neurogenesis is reported to be crucial in learning and memory in rodent experiments (Babcock et al., 2021). PA may accelerate neurogenesis, particularly that of the hippocampus (Leal-Galicia et al., 2019). The increased brain volume may thus be due to enhanced neurogenesis. However, human evidence, particularly that from older adults, is scarce.

### White Matter Integrity

White matter plays a crucial role in cognition by connecting different brain regions to enable efficient signal transmission. White matter in adult brains exhibits plasticity involving myelin formation and remodeling (Sampaio-Baptista and Johansen-Berg, 2017). Observational studies suggest that higher PA is associated with better white matter integrity as measured by diffusion tensor imaging on magnetic resonance imaging (MRI; Buchman et al., 2018; Franchetti et al., 2020; Wolf et al., 2020). Several interventional studies have found evidence of improved white matter integrity (Voss et al., 2013; Burzynska et al., 2017; Clark et al., 2019; Colmenares et al., 2021), but another study failed to show an effect (Venkatraman et al., 2020).

### Small Vessel Disease

Small vessel disease is represented by white matter lesions (WMLs). T2-weighted or fluid-attenuated MRI images visualize WMLs as diffuse high-signal areas. WMLs have been linked to cognitive impairment (Alber et al., 2019). Although the reported results are not in complete agreement, several cross-sectional and longitudinal studies have shown that PA is associated with fewer WMLs (Torres et al., 2015; Moon et al., 2018). However, no relevant interventional studies have been reported.

### Stroke

A systematic review concluded that PA reduced stroke risk (Wendel-Vos et al., 2004). Therefore, PA may contribute to reducing the risk of vascular cognitive impairment.

## Physiological Mechanisms

### Brain-Derived Neurotrophic Factor

Brain-derived neurotrophic factor is a neurotrophin that influences neuronal survival, differentiation, synapse generation, and long-term potentiation (Phillips et al., 2014). Decreased BDNF level is associated with neuropathological conditions, including mild cognitive impairment (MCI; Shimada et al., 2014). The blood concentrations of BDNF decrease with aging, and there is a strong correlation between the brain and blood levels of BDNF (Knaepen et al., 2010). A recent systematic review concluded that PA interventions increased the plasma level of BDNF in individuals with MCI or AD dementia (Ruiz-González et al., 2021). A pair of studies also showed that exercise increased plasma BDNF levels in cognitively normal older people (Müller et al., 2017; Rehfeld et al., 2018). The representative studies are summarized in Table 3.

**TABLE 3:** BDNF findings associated with PA levels.

	**Participants**	**Study design**	**Finding**
**Systematic review for interventional studies**			
Ruiz-González et al. (2021)	135 (73 experimental) MCI or AD from 5 studies	Systematic review of RCTs	PA interventions increased plasma BDNF
**Interventional**			
Müller et al. (2017)	52 (26 experimental) healthy older adults (mean age 68.3)	90-min dance twice a week for 18 months	Significant increase in BDNF in dance group
Rehfeld et al. (2018)	52 (26 experimental) healthy older adults (mean age 68.3)	90-min dance twice a week for 6 months	Significant increase in BDNF in dance group

*AD, Alzheimer’s disease; BDNF, brain-derived neurotrophic factor; MCI, mild cognitive impairment; PA, physical activity; RCTs, randomized controlled studies.*

Although the precise cause of the exercise-associated plasma BDNF increase has not yet been fully elucidated, some studies have shown increased BDNF in samples from the internal jugular vein after acute training (Rasmussen et al., 2009) and chronic regular training (Seifert et al., 2010), possibly suggesting that exercise-associated BDNF may have a central origin. Rasmussen et al. (2009) estimated that 70%–80 of circulating BDNF was derived from the brain and that the remaining levels were derived from peripheral sources. Thus, the question of whether circulating BDNF levels reflect those in the human brain warrants further study.

### Insulin-Like Growth Factor 1

Circulating insulin-like growth factor 1 (IGF-1) passes through the blood–brain barrier, exerts neuroprotective effects, and induces synaptic plasticity (Sonntag et al., 2005). The upstream IGF-1 signaling pathway also induces BDNF expression (Yan et al., 2011). Interventional trials demonstrated that aerobic exercise increased plasma IGF-1 in older adults (Kang et al., 2020), including those with MCI (Baker et al., 2010) and AD (Stein et al., 2021). A systematic review concluded that resistance training also increased IGF-1 levels in participants, including older adults (Jiang et al., 2020).

### Blood Flow

Cerebral blood flow decreases with aging and may be associated with cognitive decline (Tarumi and Zhang, 2018). This reduced blood flow may reflect a decreased cerebral metabolic rate (Marchal et al., 1992). Increased sympathetic nervous activity and impaired vasodilation may also contribute to the age-associated decline in cerebral blood flow. Cardiac output tends to decline with aging and may also be associated with reduced blood flow in the brain (Tarumi and Zhang, 2018). Regular exercise may help to counteract the effects of these aging-associated changes in cerebral blood flow.

Angiogenesis may also be involved in the ability of PA to increase blood flow (Swain et al., 2003). Vascular endothelial growth factor (VEGF) is an important regulator of angiogenesis, and several animal studies have shown increased VEGF levels with physical exercise. However, studies in humans have been inconclusive (Vital et al., 2014). Observational studies have found that sedentary older adults had lower cerebral blood flow than active older adults (Rogers et al., 1990; Thomas et al., 2013; Knight et al., 2021; Li et al., 2021). Recently, a small randomized controlled trial involving a 1-year aerobic exercise intervention reported increased cerebral blood flow in the exercise group (Tomoto et al., 2021).

## Anti-Neurodegenerative Mechanisms

### Amyloid β and Tau

Alzheimer’s disease is a major neurodegenerative disease and is the leading cause of dementia. The main pathological features of AD are accumulation of amyloid β (Aβ; senile plaques) and intracellular accumulation of hyper-phosphorylated tau (neurofibrillary tangles). PA has been associated with reduced risk of AD (Barnes and Yaffe, 2011). Extensive basic research results suggest that exercise may be associated with increased Aβ clearance (Vecchio et al., 2018), reduced Aβ production (Adlard et al., 2005), enhanced tau degeneration, and decreased tau phosphorylation (Brown et al., 2019). While animal studies have established that high PA leads to better amyloid profiles (Brown et al., 2019), human studies investigating the effects of PA on brain pathologies have been very difficult to do. However, recent advances in AD-related biomarkers are enabling research into the effects of PA on AD pathologies in human subjects.

Although cerebrospinal fluid biomarkers and amyloid PET imaging are the most frequently applied modalities in this field, the applicability of plasma biomarkers is gradually improving. Many observational studies involving measures of Aβ in cerebrospinal fluid (Liang et al., 2010; Brown et al., 2013, 2017; Law et al., 2018), plasma (Stillman et al., 2017), and PET (Head et al., 2012; Brown et al., 2013, 2017; Jeon et al., 2020; Treyer et al., 2021) have reported better profiles of amyloid-related biomarkers in active people but inconsistencies remain (de Souto Barreto et al., 2015; Jeon et al., 2020; Palta et al., 2020; Stojanovic et al., 2020). The representative studies are summarized in Table 4.

**TABLE 4:** Amyloid β findings associated with PA levels.

	**Participants**	**Study design**	**Modality**	**Finding**
**Observational**				
Brown et al. (2013)	546 cognitively healthy older adults (mean age 69.6)	Cross-sectional	PET and plasma Aβ	Lower plasma Aβ1-42/1-40 and brain amyloid observed in participants reporting higher PA levels
Law et al. (2018)	85 cognitive health older adults (mean age 64.3)	Cross-sectional	CSF Aβ	Engagement in moderate PA associated with higher Aβ42
Liang et al. (2010)	69 older adults (age 55–88)	Cross-sectional	PET	Active individuals who followed exercise guidelines had significantly lower Pittsburgh Compound-B binding
Brown et al. (2017)	139 presymptomatic mutation carriers for familial AD	Cross-sectional	CSF Aβ and PET	Individuals with low PA levels had higher mean levels of brain amyloid compared with those with high PA levels on PET but no difference in CSFAβ
Stillman et al. (2017)	149 cognitively normal older adults (mean age 83)	Longitudinal for 9–13 years	Plasma Aβ	Higher baseline PA levels associated with lower levels of plasma Aβ in subsequent assessments
Jeon et al. (2020)	287 cognitively normal older adults (mean age 72)	Cross-sectional	PET	Midlife cognitive activity not related to Aβ deposition
Treyer et al. (2021)	49 cognitively normal older adults (mean age 87.8, range 84–94 years)	Cross-sectional	PET	Higher self-reported PA in the last year associated with lower Aβ load
Head et al. (2012)	201 cognitively normal adults (mean age 65)	Cross-sectional	PET	Sedentary lifestyle associated with higher Aβ deposition
Stojanovic et al. (2020)	276 cognitively normal older adults (age 55–88; 95 for CSF and 181 for PET)	Longitudinal for 10 years	CSF Aβ and PET	Baseline PA did not impact longitudinal change in Aβ in CSF or on PET
Palta et al. (2020)	326 community-dwelling older adults (mean age: 76)	Cross-sectional	PET	Self-reported higher mid- and late-life leisure-time PA not associated with amyloid burden
de Souto Barreto et al. (2015)	271 older adults with normal or mildly impaired cognition (mean age 74.7)	Cross-sectional	PET	PA not significantly associated with Aβ deposition
**Interventional**				
Moniruzzaman et al. (2020)	MCI and AD population	Systematic review of 18 RCTs	CSF and plasma Aβ, and PET	AD pathological markers rarely investigated and the results inconclusive; most studies had relatively small sample size and limited duration

*Aβ, amyloid β; AD, Alzheimer’s disease; CSF, central spinal fluid; PA, physical activity; MCI, mild cognitive impairment; PET, positron emission tomography.*

Observational studies, mainly retrospective in nature, have suggested that individuals with higher PA tend to have biomarker profiles indicative of lower Aβ deposition in the brain (Frederiksen et al., 2019). However, interventional studies with AD biomarkers are rare and have had relatively small sample sizes. Most obtained inconclusive results, although some showed favorable effects (Moniruzzaman et al., 2020). More studies are warranted.

Researchers have investigated the effects of PA on tau-related biomarkers. Some cross-sectional studies involving cerebrospinal fluid biomarkers (Liang et al., 2010) and tau PET (Brown et al., 2018) showed lower tau profiles in participants with high PA levels, but a longitudinal study did not find an interaction between PA and tau over time.

### Oxidative Stress

Acute exercise increases oxidative stress, but regular PA is expected to regulate the cellular redox state of the brain, and PA-induced redox adaptation may contribute to the neuroprotective effects of PA (Radak et al., 2016). Animal studies showed that PA protected the brain from oxidative stress (Radák et al., 2001; Radak et al., 2006). A systematic review concluded that antioxidant indicators tend to increase and pro-oxidant indicators tend to decrease after resistance training in humans (de Sousa et al., 2017). However, another systematic review found no effects of resistance training on molecular oxidation and antioxidant capacity markers (Cuyul-Vásquez et al., 2020).

Nonetheless, studies of individuals with dementia or MCI are rare. Only Jensen et al. (2019) have measured the plasma level of 8-isoprostane, an oxidative stress marker, in AD participants before and after an exercise intervention and reported only non-significant changes.

### Inflammation

Inflammation is one of the major mechanisms of neurodegeneration (Onyango et al., 2021). PA may have anti-inflammatory potential (Mee-Inta et al., 2019). Cross-sectional observational studies have reported lower levels of inflammatory markers (IL-6 and C-reactive protein) in active older adults (Reuben et al., 2003) and determined that active women had lower levels of the plasma inflammatory biomarker TNF-α (Castells-Sánchez et al., 2021). Another observational study also found low levels of IL-6, C-reactive protein, and TNF-α (Colbert et al., 2004).

Plasma IL-6 levels decrease in response to aerobic exercise in both MCI (Nascimento et al., 2014) and AD (Abd El-Kader and Al-Jiffri, 2016). In addition, levels of C-reactive protein, a representative inflammatory marker, decrease in response to exercise interventions (Muscari et al., 2010; Alghadir et al., 2016). A systematic review concluded that exercise was associated with a decrease in C-reactive protein levels regardless of age or sex (Fedewa et al., 2017). Another recent systematic review of exercise interventions in people with MCI or dementia reported that exercise significantly decreased the levels of IL-6 and TNF-α (Huang et al., 2021).

## Functional Mechanisms

### Functional Connectivity

Functional MRI enables functionally connected brain regions to be identified by measuring simultaneous activations *via* the blood oxygen level-dependent (BOLD) signals of spatially distinct regions. A systematic review by Li et al. (2017) suggested that aerobic exercise increases functional connectivity in the default mode network, which is associated with memory and abstract thinking. Recently, Bray et al. (2021) reviewed the effect of physical exercise on functional brain network connectivity in older adults with and without cognitive impairment and concluded that physical exercise increases functional brain network connectivity.

### Allocation of Neuronal Circuits

Physical activity might somehow help to recruit new neuronal circuits that would be involved in processing tasks. An interesting fMRI study suggested that 6-week dance exercise training led to the involvement of the motor-related network during highly cognitive-demanding memory tasks, possibly as a compensatory mechanism. Exercise may accelerate the involvement of new networks in the cognitive process (Ji et al., 2018).

Event-related potentials are generated in response to specific events or stimuli such as audio sound. Studies involving the P3 component, the major focus of studies of event-related potentials, showed that exercise increases the amplitude of P3, possibly suggesting that exercise enhances the allocation of neural resources as part of a compensatory mechanism (Chang et al., 2015). However, the results are controversial (Gajewski and Falkenstein, 2018; Alatorre-Cruz et al., 2020).

### Neuronal Efficiency

Exercise may increase neuronal efficiency. An fMRI study demonstrated that an exercise group had lower BOLD signals in the hippocampus and para-hippocampal gyrus compared with a non-exercise group during a memory-encoding task. Because the BOLD signal reflects brain activity in a specific region, lower BOLD signals during a certain task mean that the task was performed with less burden, which probably reflects higher neuronal efficiency in the hippocampus and para-hippocampal gyrus in the exercise group (Friedl-Werner et al., 2020). Another trial also showed that a 12-week exercise intervention reduced neuronal activation in several brain regions, including the prefrontal cortex, during a memory task (Nishiguchi et al., 2015).

### Synaptic Plasticity

Synaptic plasticity (long-term potentiation and long-term depression) is a biological model for learning and memory processes. Animal studies have demonstrated that PA controls synaptic plasticity (Bettio et al., 2019). Synaptic plasticity can be non-invasively measured in humans *via* a combination of transcranial magnetic stimulation and recording of motor-evoked potentials. A recent study reported that PA was associated with long-term depression-like neuroplasticity in older adults (Smith et al., 2021).

## Discussion

Numerous studies have established the beneficial effects of PA on brain health. Here, we attempted to comprehensively review the potential mechanisms underlying the effects of PA on brain health. Elucidation of the mechanisms may help to establish the optimal interventional approach in terms of therapeutic effects or even lead to the development of therapeutic mimics.

Increasing PA is a relatively safe and cheap way to maintain health, including brain health. However, sedentary people often struggle to modify their behavior. Clear messages explaining the intensity and frequency of exercise required to protect brain health may help motivate them to change their behavior and lifestyle. Moreover, improved understanding of the mechanisms of the effects is expected be important for behavioral change. People may want to know how PA works, and a clearer understanding of the mechanisms involved could help encourage behavioral change.

**FIGURE 1:**
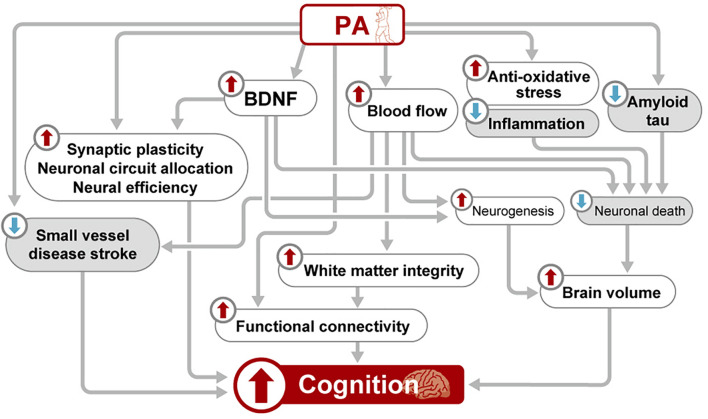
Schematic view of the mechanisms underlying the beneficial effects of PA on brain health. BDNF, brain-derived neurotrophic factor; PA, physical activity.

Several mechanisms have been summarized in the current review. Each mechanism is speculated to exert its effect both independently and interrelatedly (Figure 1). However, evidence linking these mechanisms and elucidating their interrelationship is largely lacking.

The most intensively studied topic and the one with the most accumulated evidence regarding the impact of PA on brain health is the increase in blood BDNF levels. However, the source of the BDNF production induced by PA is inconclusive, so it remains unclear whether the increase of BDNF in the blood actually reflects the increase of BDNF in the brain. Moreover, the underlying mechanisms by which increased BDNF affects human brain function have not been completely elucidated. BDNF is presumed to be associated with neuronal survival and neurogenesis, and it may also preserve white matter structure (Miyamoto et al., 2014). BDNF may play further roles in preserving brain health such as by modulating neuronal/synaptic activity and blood flow (Santhanam et al., 2010; Leal et al., 2015). From a practical point of view, more information is needed to determine the optimal exercise protocol in terms of intensity and frequency. The effects of PA on brain volume seem to be more or less established. However, whether PA merely decreases aging-related brain volume loss or even increases brain volume is still unclear, and this may be relevant to the issue of whether PA only prevents neurodegeneration or enhances neurogenesis in human brain. Also, it is largely unknown how long the effects of PA on brain volume last.

More importantly, the mechanisms by which PA affects human brain volume remain to be elucidated. BDNF may at least partly help to support brain volume preservation, possibly through neuronal survival and/or neurogenesis. However, it is not yet clear whether the preservation/increase of brain volume is due to the preservation/increase of the number of neurons in humans. There is room for further research. It also remains to be elucidated how an increase in BDNF might contribute to the preservation of brain volume in humans. The relative contribution of neurogenesis and prevention of neuronal death to the preservation of brain volume is not yet fully understood. Although BDNF has effects on neuronal preservation, PA may also counteract neurodegeneration through pathways different from those of BDNF, including antioxidative and anti-inflammatory effects.

Epidemiological evidence indicates that PA reduces the risk of clinically diagnosed AD (Buchman et al., 2012). Theoretically, several possibilities, which are not mutually exclusive, may be involved in the ability of PA to reduce the risk of a clinical presentation of AD: (1) PA directly modifies amyloid and tau metabolism; (2) PA reduces neurodegeneration provoked by the amyloid cascade; and (3) PA enhances brain function.

Some studies of AD-related biomarkers suggest that PA may directly modify AD-related pathologies. However, taken together, the results of these studies are as yet inconclusive. In particular, insufficient evidence has been accumulated from randomized controlled studies (Table 4). As discussed above, PA may have antioxidative and anti-inflammatory effects, and these effects may help neurons to survive in the neurodegenerative pathway initiated by Aβ, somewhere downstream of the amyloid cascade. It should be clarified whether PA directly modifies the amyloid cascade or exerts neuroprotective effects downstream of the cascade. Moreover, some studies have suggested that PA may have effects on cognition that are independent of pathological changes. Here, we reviewed several potential mechanisms distinct from neurodegeneration modification or ischemic pathologies. An increase in blood flow may be critical to the modification of brain function, with other contributions coming from maintained white matter microstructure and connectivity. Neuronal compensation, neuronal resource allocation, and neuronal efficiency are relatively under-studied areas and warrant further research. Also, studying a combination of several types of biomarkers—amyloid-related, tau-related, and neurodegeneration-related—might help to elucidate how PA prevents AD. Prospective interventional studies involving the combination of several biomarkers would be valuable.

To conclude, numerous studies have been conducted in this field and a substantial amount of evidence has been accumulated. Two points have been clarified so far, namely, the association of PA with increased blood levels of BDNF and its association with brain volume preservation. However, much remains to be elucidated. For example, the mechanism by which circulating BDNF affects the brain as well as the association between increased BDNF levels and neurogenesis in people are unclear. Although animal studies have demonstrated that BDNF increases neurogenesis in the hippocampus, the contribution of BDNF to neurogenesis in the context of human brain health, especially in older adults, has yet to be clarified. In addition, even though the effects of PA on AD-related pathologies have been extensively studied, the research results are inconsistent, and so it remains unclear whether PA is associated with less AD-related pathologies. Many of the studies conducted in this field have been small in scale and have employed cross-sectional designs. Larger longitudinal studies or RCTs are needed to understand the associations between PA and AD-related pathologies. In this review, many other potential mechanisms were discussed. Although these mechanisms are interesting and could possibly be correct, at this point clear evidence is lacking. It would be very important for people, especially those who lead sedentary lives, to know how PA affects their brain because such knowledge has the potential to motivate them to increase their PA. Also, elucidation of the mechanisms may lead to the development of effective exercise programs as well as methods for efficiently monitoring the benefits. A more in-depth and clearer understanding of the mechanisms underlying the effects of PA on brain health is therefore needed. The advancement of this research field is eagerly expected.

## Author Contributions

HU designed the review and wrote the draft. TS and HA contributed the content and edited the draft. All authors contributed to the article and approved the submitted version.

## Conflict of Interest

The authors declare that the research was conducted in the absence of any commercial or financial relationships that could be construed as a potential conflict of interest.

## Publisher’s Note

All claims expressed in this article are solely those of the authors and do not necessarily represent those of their affiliated organizations, or those of the publisher, the editors and the reviewers. Any product that may be evaluated in this article, or claim that may be made by its manufacturer, is not guaranteed or endorsed by the publisher.
